# A Review on Porous Polymeric Membrane Preparation. Part I: Production Techniques with Polysulfone and Poly (Vinylidene Fluoride)

**DOI:** 10.3390/polym11071160

**Published:** 2019-07-08

**Authors:** XueMei Tan, Denis Rodrigue

**Affiliations:** 1 College of Environment and Resources, Chongqing Technology and Business University, Chongqing 400067, China; 2 Department of Chemical Engineering, Laval University, Quebec, QC G1V 0A6, Canada

**Keywords:** porous polymeric membranes, polysulfone, poly(vinylidene fluoride), preparation, morphology, performance

## Abstract

Porous polymeric membranes have emerged as the core technology in the field of separation. But some challenges remain for several methods used for membrane fabrication, suggesting the need for a critical review of the literature. We present here an overview on porous polymeric membrane preparation and characterization for two commonly used polymers: polysulfone and poly (vinylidene fluoride). Five different methods for membrane fabrication are introduced: non-solvent induced phase separation, vapor-induced phase separation, electrospinning, track etching and sintering. The key factors of each method are discussed, including the solvent and non-solvent system type and composition, the polymer solution composition and concentration, the processing parameters, and the ambient conditions. To evaluate these methods, a brief description on membrane characterization is given related to morphology and performance. One objective of this review is to present the basics for selecting an appropriate method and membrane fabrication systems with appropriate processing conditions to produce membranes with the desired morphology, performance and stability, as well as to select the best methods to determine these properties.

## 1. Introduction

Membrane technologies, as efficient separation techniques, have been widely applied in several areas, especially for water treatment (drinking water and wastewater treatment) including disinfection, distillation or media filtration [1,2,3,4,5], pharmaceutical and biotechnology industries such as drug release [6,7], food industries such as membrane fractionation of milk [8,9], as well as renewable energy storage and transformation processes such as conducting membranes in artificial photosynthetic systems and membranes for redox flow battery [10,11,12].

While membranes have shown significant performances in different applications, the drive to improve the membrane success requires membranes with better materials and performances. The combination of properties such as permeability, selectivity, fouling resistance, chemical and thermal stability, low cost and easy manufacturing should lead to improved characteristics. Firstly, polymeric membranes perform well in commercially available membrane applications. The most commonly used engineering or commodity polymeric membranes include polysulfone (PS), poly(vinylidene fluoride) (PVDF), polyethylene (PE), polydimethylsiloxane (PDMS) and other materials [13]. Secondly, a careful control of the morphology must be made [14,15,16]. Homogeneity and a well-controlled microporous structure both define the success of a membrane.

The purpose of this paper is to critically review the methods to control the morphology (microporous structure) of polymeric membranes. To start, membrane characterization is presented including morphological and separation performances, followed by a review of two commonly used membrane polymers (PS and PVDF). Then, five different methods for membrane fabrication are introduced: non-solvent induced phase separation (NIPS), vapor-induced phase separation (VIPS), electrospinning, track etching and sintering. Each method will be described in detail with respect to their key factors in manufacturing, providing emphasis on the relationships between processing (methods and conditions) and morphology control. Finally, to produce membranes with the desired morphology, performance and stability, comparisons between various preparation methods are presented.

## 2. Morphological and Performance Characterization of Membranes

### 2.1. Membrane Morphology

#### 2.1.1. Morphological Parameters

In general, the most important morphological parameters for a membrane are gravimetric porosity, pore size, pore size distribution, tortuosity, surface roughness, molecular weight cutoff and thickness. Each parameter is defined next.

Gravimetric porosity is a measure of the total amount of void in a membrane. It represents the ratio between the membrane density and the density of the neat matrix [16].

Pore size represents the dimensions of the pores, which are channels of a variable cross-section. The distance between two opposite pore walls is used as the pore size for simple geometries (diameter of cylindrical pores for pore size >2 nm, width of slit-shaped pores for pore size <2 nm). If the pores have irregular shapes, some averaging is made to report an average pore size. In general, an equivalent spherical shape is assumed, but other representations have been proposed such as cage-like/spherical mesoporous structure, non-spherical particles, etc. [17,18,19,20,21]. Since the pores do not all have the same size and/or geometry, some statistical analysis must be performed using a model such as nonlinear optimization and Monte Carlo integration, to get a representation of the pore size distribution [22].

Tortuosity is defined as the reciprocal of the average ratio between the straight distance connecting two points (membrane thickness) and the actual path length inside the porous medium (on average). This parameter provides some information on the difference between the real internal pore shape and an ideal structure [16,23].

Surface roughness is quantified by the deviations in the direction of the normal vector of an actual surface from its ideal geometric flat shape and dimensions [24].

The molecular weight cutoff (MWCO) refers to the lowest molecular weight solute (in dalton) for which 90% of the solute is retained by the membrane, or the molecular weight of the molecule that is 90% retained by the membrane [25].

The thickness represents the distance between both surfaces (top and bottom or front and back) of a membrane.

**Figure 1:**
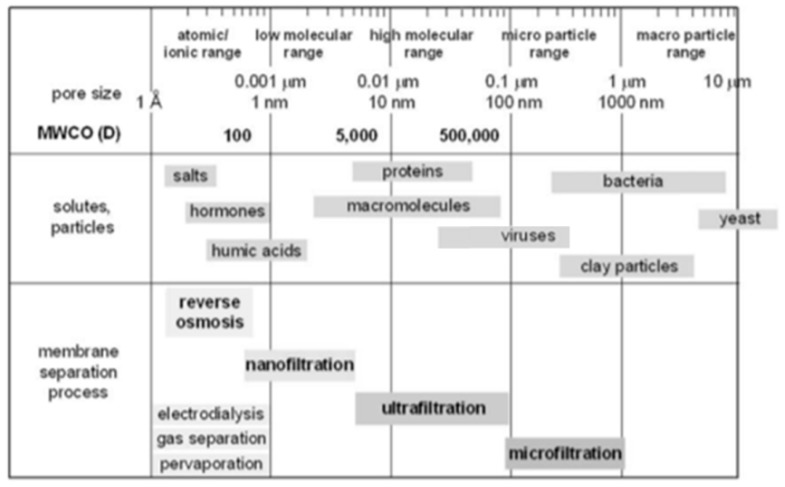
Membrane separation processes, pore sizes, molecular weight cut-off (MWCO) and typical solutes and particles sizes, adapted from references [26,27].

Membranes can also be divided into four types depending on their application: microfiltration, ultra-filtration, nano-filtration and osmosis. The classification corresponds to their average pore sizes which are in the range of 50–500 nm, 1–50 nm, ≤1 nm and 0.3–0.6 nm, respectively. Figure 1 shows that based on the different pore type, an appropriate membrane can be selected according to the characteristics of the target species to separate [26,27]. From a structural point of view, a membrane can be defined as asymmetric if the pore size distribution is not uniform across the membrane thickness, while symmetrical membranes are uniform. For asymmetric membranes, a very thin dense surface layer is present acting as a functional layer on top of a porous layer with a specific pore diameter, and a support layer below. Today, most industrial applications use asymmetric membranes and their manufacturing lays down the foundation of membrane separation technology.

#### 2.1.2. Morphological Characterization

Since membrane performances directly depend on their morphology (pore size and distribution), morphology control is the key factor in membrane fabrication. To characterize the morphological parameters, several techniques are available. To start, microscopy is used including scanning electron microscopy (SEM), atomic force microscopy (AFM), confocal scanning laser microscopy (CSLM) and transmission electron microscopy (TEM) [16]. The main limitations of these methods are long sample preparation time and only a 2D (surface) analysis is performed. Today, faster and more complete techniques can give a 3D (volume) analysis like X-ray computed microtomography (micro-CT), nuclear magnetic resonance (NMR), spin-echo small-angle neutron scattering (SESANS) and magnetic small-angle neutron scattering (MSANS) [28,29].

### 2.2. Performance Characterization

Generally, the membrane performances can be evaluated by their productivity (rate) and separation ability (selectivity). Parameters like flux (*J*), permeability (*P*) and permeance (*P*’) play essential roles in the evaluation of the membrane productivity, while efficiency (separation performance) is determined by the selectivity (α) and separation (β) factors [30,31]. The flux can be calculated as:

*J* = *Q*/(*A t*) (1)

where *Q* is the total weight of the permeate passing through the membrane, *t* is a specific time period and A is the effective surface area.

Permeability (*P*) represents the trans-membrane permeation flux of a particular component which can be approximated by:

*P* = *k*_sm_*k*_m_ λ/(2*k*_m_ + *m k*_ms_) (2)

where *k*_m_ is a diffusion constant for the membrane, *k*_sm_ is a diffusion constant through the solution-membrane interface, *k*_ms_ is a constant for diffusion through the membrane-solution interface, m is a constant related to the membrane thickness and λ is the minimum distance between the equivalent positions.

On the other hand, permeance (*P*’) represents the permeability of a component across the membrane per unit of thickness (*l*) to give:

*P*’ = *P*/*l* (3)

Selectivity (α_ij_) represents the ratio between the permeability of each component (*i* and *j*). The value can be expressed using mass or mole as:

(α_ij_)_mass_ = (*P*_i/_*P*_j_) = ((*P*_i/_*l*)/(*P*_j/_*l*)) (4)

(α_ij_)_mole_ = (*M*_i/_*M*_j_) (α_ij_)_mass_ (5)

where indices *i* and *j* refer to each component, while *M* is the molar mass.

The separation factor (β_ij_) represents the relative fraction of each components in the permeate (*y*) and feed (*x*) to give:

β_ij_ = ((*y*_j/_*y*_i_)/(*x*_j/_*x*_i_)) (6)

## 3. Materials

The “Global Market Study on Membrane Separation: Water & Waste Water Segment to Witness Highest Growth by 2019” reported that the global membrane separation market was valued at US$ 19.0 billion in 2012 and is expected to grow at a compound annual growth rate (CAGR) of 10.8% from 2013 to 2019, to reach an estimated value of US$ 39.2 billion in 2019. Since polymer membranes are the most widely used membranes for separation processes due to their low cost, chemical stability and mechanical strength [16], the world market for polymer membrane is estimated to keep growing as well. Here a focus is made on PS and PVDF as the most widely used polymers for separation membranes.

### 3.1. PS Membrane

PS is an important thermoplastic used for the fabrication of polymer membranes [16,32]. This is related to the structure of its repeating unit [OC_6_H_4_OC_6_H_4_SO_2_C_6_H_4_]_n_ providing excellent properties such as thermal stability (150–170 °C), chemical inertness over the whole pH range, mechanical strength (fracture toughness, flexion and torsion) and processability [16,33,34,35]. Recently, several investigations focused on PS membranes for a wide range of applications such as micro-/ultra-filtration in water and wastewater treatment, membrane distillation, membrane oxygenators, gas separation, pervaporation, separator for lithium ion battery, plasma separator and support for composite membranes [16,34,36,37,38,39,40,41].

Two methods are known as effective techniques for the preparation of PS membranes: phase inversion and electrospinning [34,42,43,44,45]. Each one is described later.

### 3.2. PVDF Membranes

PVDF is one of the most widely used semi-crystalline polymers for membrane preparation. The polymer is composed of a CF_2_–CH_2_ repeating unit. Compared to other commercial polymers, PVDF has some outstanding properties such as high mechanical strength, thermal stability, chemical resistance and hydrophobicity [46]. Typical PVDF properties are listed in Table 1.

**Figure 2:**
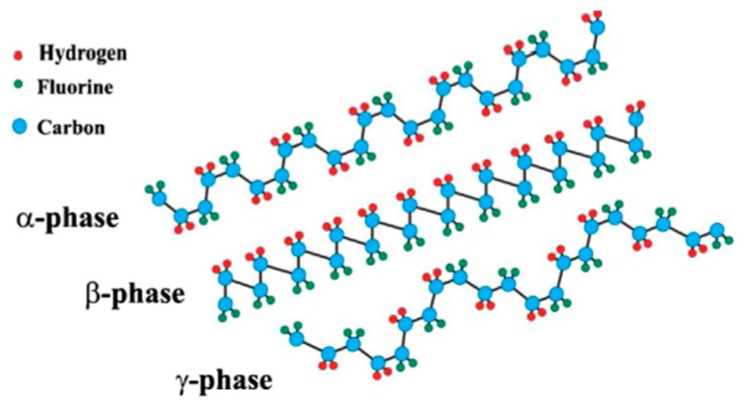
Schematic representation of the chain conformation for the α, β and γ phases of PVDF, adapted from reference [51]. [A color figure can be viewed in the online issue, which is available at wileyonlinelibrary.com].

The different spatial arrangement of the CH_2_ and CF_2_ groups along the PVDF chains generates five distinct crystalline polymorphs or forms: α, β, γ, δ and ε phases [46]. These crystal phases differ from their molecular and crystal dipole moments. For example, α-PVDF and δ-PVDF structures have the TGTG’ (T-*trans*, G-*gauche^+^*, G’-*gauche^-^*) conformation, β-PVDF has all TTTT conformation, while γ-PVDF and ε-PVDF have a TTTGTTTG’ conformation [47]. It is well known that the α-, β- and γ-phases are the most common phases of PVDF (Figure 2). The non-polar crystalline α-phase is the most favorable polymorph in membrane fabrication, which is due to it being a kinetically favorable phase, the β-phase in which the polymer chains are in the polar zigzag confirmation has the largest dipolar moment of 8 × 10^−30^ C·m [48], while the polar crystalline γ-phase also exhibits interesting electroactive properties. Furthermore, the intrinsic properties of PVDF membrane are significantly affected by the polymorphism [48]. For ultrafiltration/microfiltration applications, various polymorphs can cause different interactions between the PVDF membrane and the solution, and consequently result in the different fouling behavior. Previous works have shown that α–PVDF has better anti-fouling properties, while higher β–PVDF may improve the ability of protein binding [49]. Since the discovery of piezoelectricity in PVDF which is due to the strong electrical dipole moment of the PVDF monomer unit (5–8 × 10^−30^ C·m), a great deal of interest has been devoted to the piezoelectric response of PVDF for membrane sensors [50].

## 4. Methods of Preparation

### 4.1. Phase Inversion

Presently, most of the commercial polymer membranes are produced via phase inversion methods due to their simple processing, flexible production scales and low cost [46]. According to different desolvation mechanisms, four main types of phase inversion methods are known: NIPS, VIPS, thermally induced phase separation (TIPS) and solvent evaporation-induced phase separation (SEIPS). Among them, NIPS, VIPS and TIPS are widely applied for the production of polymer membranes [48,52], because SEIPS does not use polymers but liquid monomers for membrane formation. Asymmetric membranes are obtained by stopping the phase separation of the monomers and water with nanoparticles forming dense and jammed interfacial layers. The monomers are then polymerized to produce porous solid membranes [53].

In membrane preparation via TIPS, a polymer solution is formed at high temperature and cooled to induce phase separation and polymer solidification. The porous membranes are obtained after the extraction of the diluent [54]. TIPS was introduced in the late 1980s to fabricate PVDF microporous membranes, and the method was significantly developed recently due to the emergence of PVDF membrane contactors, as well as their wide use as microfiltration and ultrafiltration membranes [46,48,49,55,56]. To date, a few works were carried out to investigate the preparation of PS membranes via the TIPS process since PS is amorphous [54]. However, low mutual affinity between the solvent and non-solvent in TIPS results in surface pores which are difficult to control. Furthermore, the TIPS method is mainly used to prepare crystalline polymer membranes and especially suitable for semi-crystalline polymers which cannot be easily dissolved by solvents, such as polyethylene and polypropylene [57,58,59]. The next section provides a brief discussion on the preparation of PVDF and PS membranes via NIPS and VIPS.

#### 4.1.1. Non-Solvent Induced Phase Separation (NIPS)

##### NIPS Process

The NIPS reaction system uses a ternary composition, usually including a polymer, a solvent and a non-solvent. The NIPS process (immersion precipitation) starts by mixing at least a polymer and a solvent to form an initial homogeneous solution. Then, the polymer solution is cast as a thin film on a support or extruded through a die to generate the membrane shapes such as flat sheets or hollow fibers [60]. Subsequently, the material goes into a coagulation bath containing a non-solvent or a poor solvent for the polymer, and hence, phase separation takes place when the solvent exchanges into the non-solvent and precipitation occurs in the polymeric solution [16,52,60,61].

##### Porous Membranes Formation Mechanism of NIPS

**Figure 3:**
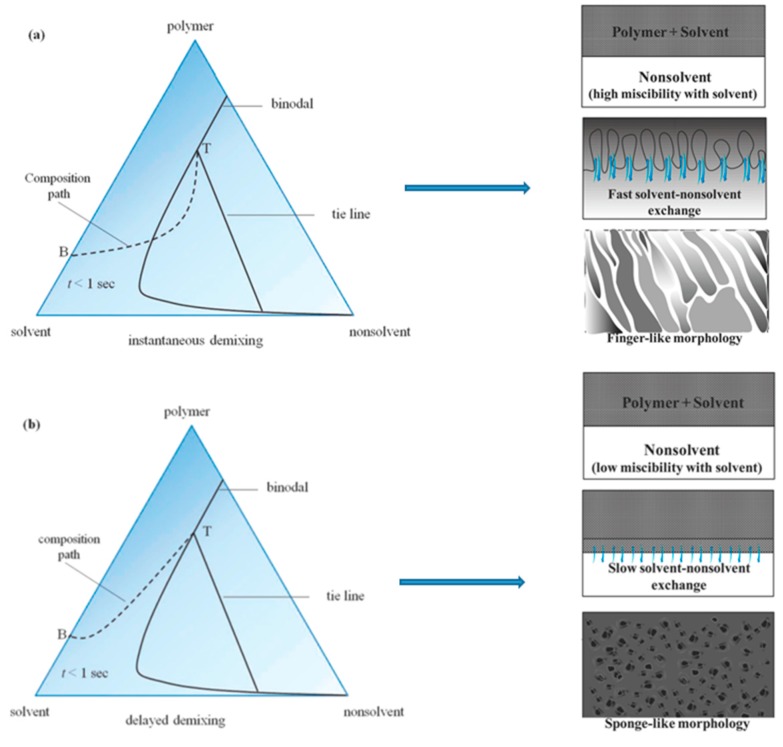
Different membrane morphologies associated to different types of demixing, adapted from reference [71]. [A color figure can be viewed in the online issue, which is available at wileyonlinelibrary.com.].

Several mathematical models of the phase inversion process were published [52,62,63,64,65,66,67,68,69]. Taking immersion precipitation as an example, liquid-liquid phase demixing plays a key role in this process. The model of Reuvers et al. [44] and Smolders et al. [70] discussed the thermodynamic aspects of instantaneous demixing and delayed demixing processes to differentiate the porous structures. Instantaneous demixing represents the solution firstly demixing followed by further changes of the polymer rich phase composition going through the glass transition. Delayed demixing represents the composition of the interface through the vitrification boundary without demixing [52,71]. As Figure 3 shows, instantaneous demixing means that the polymer precipitates very quickly after immersion in the non-solvent bath and the membrane generally shows a finger-like pore substructure, fine gravimetric porosity and thin skin layers. If the composition profile does not touch the binodal line, this type of demixing is called delayed demixing. In this case, precipitation takes long time and the membrane formation is slow. These membranes show a sponge-like substructure and a relatively dense top layer [71].

Although the theoretical models can predict the size and location of the liquid-liquid demixing gap for its influence on the pore structures, the situation is more complex as several interactions are involved between the membrane morphology and the system parameters for the immersion precipitation process [52,67,70,71,72,73,74]. Van de Witte et al. [52] found that five pore structures were obtained by immersion precipitation: unconnected latex, nodules, bicontinuous structures, cellular structures and macrovoids. Note that the thermodynamic diagrams may only predict an equilibrium process during membrane fabrication, but the process kinetics determine whether or not the thermodynamic phase transition will occur. They also determine to which extent the phase separation will take place. It should be kept in mind that non-equilibrium processes may significantly influence the pore structures during membrane formation [52]. As such, the next section presents the parameters affecting the membrane separation performance.

##### Effects of Various Parameters on NIPS

I. Solvent and non-solvent selection

For NIPS, the first step is to form a homogeneous solution by choosing the solvent to dissolve or easily disperse the polymer. Solubility parameters such as the dispersive parameter (δ_d_), polar parameter (δ_p_) and hydrogen bonding parameter (δ_h_) can help selecting the appropriate solvent-polymer systems. Furthermore, Hansen and Skaarup developed the radius of interaction of a Hansen solubility parameter sphere (R_o_) [75]. For example, the R_o_ value for PS is 8.0 MPa^1/2^ while for cellulose acetate it is 13.7 MPa^1/2^ [71]. They further defined a parameter to measure the solution affinities between polymer and solvent (R_a_) and a concept of relative energy difference (RED), where RED < 1 means the solvents are compatible with a given polymer and solubility increases when RED approaches zero. Usually, it is relatively easy for a given polymer to choose between several compatible solvents [71]. In addition, Tsai et al. [76] discussed the effect of solvent quality on membrane morphology. They found that during membrane formation, a poor solvent such as 2-pyrrolidone can be used to prepare highly porous PS membranes with interconnected pores, but a better solvent such as n-methylpyrrolidinone does not produce the same structure [60,76]. Yeow et al. [77] also found that a weak solvent produced a sponge-like porous structure while a stronger solvent led to the formation of macrovoids [78]. It is worth noting that to account for environmental concerns, ionic liquids have been selected to replace traditional organic solvents for membrane fabrication [60,79].

On the other hand, the solvent and non-solvent must be miscible. In the case of high mutual affinity (or miscibility), a more porous membrane is likely to be obtained. The low mutual affinity between the solvent and non-solvent is more likely to delay demixing. As a result, an asymmetric membrane with a dense nonporous top layer is obtained. Water is frequently used as a non-solvent because it is environmentally friendly and low cost.

In view of the important choice of the solvent/non-solvent match on the membrane structure, Table 2 lists some experimental works done on PS in different solvent/non-solvent ternary systems. It can be seen that two main solvents and non-solvent systems are used for PS membrane fabrication: dimethyl acetamide (DMAc)/water and N-methyl-2-pyrrolidone (NMP)/water. However, N,N-dimethylformamide (DMF) is also used as a solvent, while tetrahydrofuran (THF) is used as a volatile co-solvent.

For PVDF membranes, NIPS is the main technique to produce porous PVDF membranes. PVDF can be easily dissolved in common organic solvents such as N,N-Dimethylacetamide (DMAc), N,N-Dimethylformamide (DMF), Dimethylsulfoxide (DMSO), Hexamethyl phosphoramide (HMPA), N-Methyl-2-pyrrolidone (NMP), Tetramethylurea (TMU), Triethyl phosphate (TEP), Trimethyl phosphate (TMP), Acetone (Ac), DMF/Ac, methyl ethyl ketone (MEK) and Tetrahydrofuran (THF) [46,83,84]. To date, an emerging technique is to use supercritical carbon dioxide (sc-CO_2_) as the non-solvent in the preparation of PVDF (see Table 3) as well as PS membranes [60,85,86,87,88,89]. This method can be considered as an improvement of the traditional phase inversion process because it is more environmentally friendly and has lower cost (recovery). Furthermore, sc-CO_2_ can form and dry the membrane rapidly without structure collapse due to the absence of a liquid-vapor interface [46,85]. Until now, phase inversion assisted by sc-CO_2_ is limited to the membrane formation and in limited cases of drug-loaded membranes [90,91]. Future researches will focus on generating uniform distribution of nano-scale pores in the membranes.

II. Polymer solution composition

(1) Polymer concentration and properties

Since the polymer is the component forming the membrane matrix, the polymer concentration in the casting solution will influence the final membrane morphology. Typically, the higher the polymer concentration, the lower the gravimetric porosity. Furthermore, when the polymer concentration is above a certain threshold, there is not enough solvent and non-solvent exchange in the dope solution to form the pores during the phase separation and solidification process, so the membrane gravimetric porosity is reduced and permeability is lost. Some researchers showed that different initial polymer solution could result in different precipitation paths [67,71,94,95,96]. This implies that increasing the polymer concentration will increase the probability of instantaneous demixing, leading to differences in membrane morphology.

Hołda et al. [82] showed that the PS molecular weight and purity are other parameters affecting the membrane morphology and performance. They selected 10 different PS to prepare the membrane via immersion precipitation. The results showed that purification of the starting polymer can increase the membranes flux performance. Meanwhile, blending induces the formation of macrovoids, resulting in membranes showing better performances.

(2) Additives in the polymer solution

Recently, studies on additives in the polymer solution have significantly increased. Although the addition of organic or inorganic components as a third component into a casting solution makes the solvent/non-solvent system more complex, these additives can clearly influence the pore formation and structure, enhance the pore interconnectivity, as well as improve the hydrophilicity and performance of the final membrane [71].

The most common polymer additives are hydrophilic polymers, such as polyvinylpyrrolidone (PVP) and polyethylene glycol (PEG). Several researchers have reported that increasing the PVP molecular weight led to thicker membrane skin layers, while the membrane sub-layers had dense structures with fewer macrovoids and the number of finger-like macrovoids gradually disappeared [43,97,98,99,100,101]. Kim et al. [102] reported that higher PEG concentration leads to larger membrane surface pores and higher gravimetric porosity. Chakrabarty et al. [103] reported that increasing the PEG molecular weight increased the pore number and gravimetric porosity. Other studies also found that PEG can improve the membrane hydrophilicity and selectivity [101,104,105,106,107], in agreement with previous findings. Although the introduction of hydrophilic polymers can improve the membrane gravimetric porosity and permeability during the fabrication process, it has been shown that PVP or PEG are unstable in air or aqueous environments [108,109]. As a result, membrane formation via blending with amphiphilic copolymers has been extensively studied. Zhao et al. [35] reported the fabrication of a PS-based copolymer containing poly(N,N-dimethylamino-2-ethylmethacrylate) (PDMAEMA) blocks, while Yi et al. [110] further designed PS-POEM block copolymers as hydrophilic additives for the preparation of antifouling membranes. All of these results showed that the membranes surface hydrophilicity and fouling-resistance were significantly enhanced after block copolymer addition and surface zwitterionicalization. For this new research field, the migration and transformation mechanism of the copolymer additives as a third component in the casting solution should be the focus of future research.

Surfactants, such as 1,4-dioxane, diethylene glycol dimethyl ether (DGDE), acetone, γ-butyrolactone (GBL) [32], Tetronic 1107 hydrophilic surfactants (ethylenediamine tetrakis(ethoxylated-block-propoxylate) tetrol), Tween 20 (polyoxyethylene sorbitan monolaurate) [111,112] and sorbitan monooleate series (Span-20, Span-80) [113,114,115,116], have been shown to increase the affinity between the solvent and coagulant resulting in a change of the precipitation path [117]. Furthermore, smaller surfactant molecules in the casting solution can produce larger pores on the top surface and a more porous structure in the sub-layer leading to thicker membranes [111,112]. Overall, the addition of surfactants has a significant effect on the membrane structure since their presence can substantially affect the affinity between the casting solution and the coagulant.

Today, using nanoparticles as additives in the polymeric composite membrane fabrication is a cutting-edge area of membrane science. Nanoparticles, such as carbon nanotubes [118], multi-walled carbon nanotubes [119], silica nanoparticles [120,121], silver nanoparticles [122], titanium oxide nanoparticles [123] and ZnO nanoparticles [124], have been used as fillers in polymer composite membranes and the large surface area to volume ratio resulted in strong interfacial interactions between the nanoparticles and the surrounding polymer [125,126,127]. Table 4 reports on typical additives used for PS composite membranes.

III. Film casting conditions

The main film casting conditions are the composition and temperature of the coagulation bath.

Ghosh et al. [130] reported that the addition of small amounts of solvent in the coagulation bath was an effective method to prepare nonporous membranes due to the decreasing rate of mass exchange between the non-solvent and casting solution resulting in a shift from instantaneous demixing to delayed demixing [131,132]. It is worth noting that too high solvent addition into the coagulation bath would lead to the polymer concentration not being high enough (dilution effect) to form a good membrane [71,133]. Similarly, other additives can be added into the coagulation bath such as methanol and tetrahydrofuran [82], as well as isopropanol [87].

The casting temperature can also affect the solution viscosity resulting in the exchange rate varying during the phase inversion step and affecting the membrane surface and internal morphology [116]. Zheng et al. [134] reported that the size of the finger-like macrovoid increased with increasing the coagulation bath temperature.

Last but not least, none of these parameters alone is able to optimize the membrane morphology; i.e., the final polymer membrane morphology and separation performance are determined by the combined effects of all these parameters. Taking PVDF as an example, Table 5 presents the relationships between the various NIPS parameters and the membrane structure.

#### 4.1.2. Novel Morphology Control Techniques Based on NIPS

Two novel processes based on NIPS are discussed below: a combination of NIPS and block copolymer self-assembly and a combination of NIPS and TIPS [16,60].

##### Combination of NIPS and Block Copolymers Self-Assembly (SNIPS)

Recently, an emerging technique combining self-assembly and non-solvent induced phase separation (SNIPS) was reported to produce asymmetric polymer membranes with a well-ordered top layer featuring high density and uniform nanoscale pores. Usually, block copolymers are dissolved in some specific solvent mixture, which is the critical step in the assembly of copolymer micelles in the solution. Then, the polymer solution is cast into a film, controlled solvent evaporation occurs followed by a rapid exchange between the solvent and non-solvent. Finally, the polymer precipitates to produce the final nanoporous membrane. The stabilized and frozen copolymer micelles produced by the dry/wet phase separation method is the key step of SNIPS which is the direct result of the selective layer of uniform pores via self-assembly upon solvent evaporation [60,148]. In particular, several critical strategies of membrane fabrication by SNIPS are identified: the careful adjustment of the solvent quality to prepare an appropriate self-assembly pattern [149], the introduction of metal ions to form metal-polymer complexation [150,151], the addition of low-quality solvent to stabilize the micelle core blocks in the casting solution [152], as well as the addition of volatile components to create a short solvent evaporation window before NIPS [148,152].

To increase the production scale of SNIPS membranes from the laboratory to a commercial production, some researchers elucidated the mechanisms of the self-assembly process and final assembled morphology [153,154,155,156]. For example, Caicedo-Casso et al. [148] used three different A-B-C triblock terpolymer chemistries of similar molar mass to fabricate isoporous asymmetric membranes via SNIPS. The results showed that the formation of a viscoelastic film typical of asymmetric membranes was strongly dependent on solvent evaporation, with polyisoprene-b-polystyrene-b-poly(4-vinylpyridine) (ISV) solutions displaying the greatest solution viscosity and fastest strength development combined with the greatest strength magnitude of the evaporation-induced viscoelastic film. Phillip et al. [157,158] reported the effect of the solvent evaporation rate and time on the selective layer of a SNIPS membrane. Their results showed that fast evaporation was necessary for perpendicularly oriented cylinders and the final copolymer layer thickness was 4 μm. But further optimization must include the introduction of more parameters to account for convection, shear stresses and film deformation [148]. This is necessary to develop a completely scalable membrane manufacturing process.

##### Combination of NIPS and TIPS

Recently, some researchers combined NIPS and TIPS (N-TIPS) to obtain a tailored surface pore structure. The main objective of N-TIPS is to produce high gravimetric porosity on the membrane via NIPS and uniform micopores on the support side via TIPS.

One approach is featured as a NIPS-dominated process. Matsuyama et al. [159] first bridged the gap by applying TIPS immediately after the immersion. They used water having low mutual affinity with cyclohexanol (diluent) and methanol, but high affinity as the non-solvent. As a result, they obtained a thin skin layer or macrovoids near the top surface due to NIPS and smaller pores near the bottom surface due to TIPS. For PVDF membranes, Jung et al. [160], Hassankiadeh et al. [161], Xiao et al. [162] and Wu and Sun [163], modified TIPS by using a water-soluble poor solvent (TIPS solvent) as the diluent. However, this method may form some undesired membrane morphology such as a dense layer with scarce pores caused by the fast outflow of diluents leading to high permeability loss [159,163,164]. Conversely, the finger-like macrovoids in the bulk structure near the surface layer due to a large concentration gradient between the solvent and non-solvent [48,162] can significantly decrease the membranes mechanical properties [46,132].

At the same time, some researchers investigated high-temperature NIPS by using common NIPS solvent or by adding a water-soluble good solvent into the diluent [165,166,167,168]. Notably, such dope systems can only be operated at a relatively low temperature because of the low boiling point of the common NIPS solvents [166]. As a result, the membranes fabricated through this method usually exhibit good mechanical properties due to the reduced temperature gradient leading to sufficient time for the polymer crystallization, although non-uniform pore size distribution and macrovoids induced by NIPS still exist [165,166].

**Figure 4:**
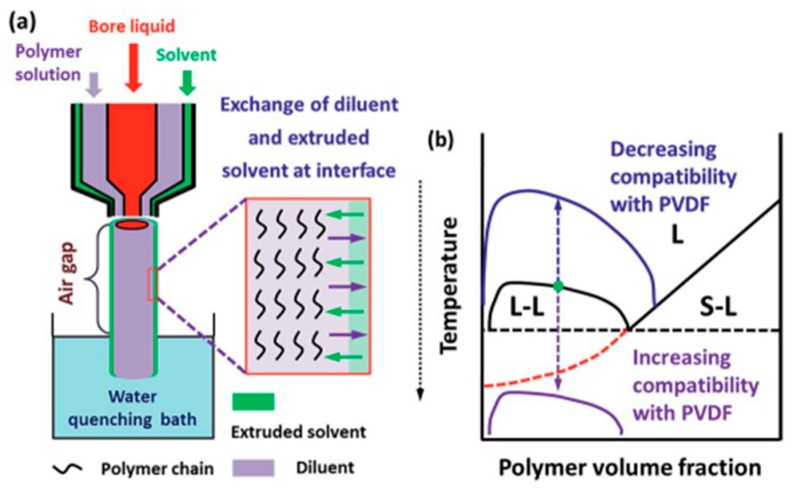
Interfacial behavior of the diluent or polymer chains (**a**) and the change in the phase separation (**b**) during membrane formation in the presence of extruded solvents having different compatibilities with the polymer at the outer surface. S and L are the solid and liquid, respectively. Adapted from reference [171]. [A color figure can be viewed in the online issue, which is available at wileyonlinelibrary.com.].

The third approach of N-TIPS is co-extrusion by extruding a NIPS coating solution or a solvent on the outer layer using a triple-orifice spinneret. Lee et al. [169] successfully prepared PVDF dual-layer hollow fiber membranes with porous layers by this method. The results showed that micron-sized holes in the spherulite structure were formed by introducing non-solvent additives into the TIPS dope solution. Furthermore, this morphology significantly increased the water flux due to increased membrane gravimetric porosity. Fang et al. [170,171] tailored both the surface pore size and sub-layer structures of PVDF membranes prepared by N-TIPS with a triple orifice spinneret. The results showed that the diffusion of extruded solvents having good compatibility with PVDF into the polymer solutions changed the phase separation mechanism from L-L to S-L, followed by L-L phase separation (see Figure 4), resulting in the formation of a novel composite-like structure (spherulites connected by the bicontinuous network structure) in the membrane sub-layer. However, this approach requires coordination between the dope mixtures and coating solution during the spinning, resulting in the whole process being relatively complex and the operating conditions being critical. Moreover, the membranes produced could exhibit some undesired delamination and irregular surface structure due to the asynchronous curing process of the dual layer [172,173].

#### 4.1.3. Vapor-Induced Phase Separation (VIPS)

##### Introduction

In 1918, Zsigmondy and Bachmann first reported the vapor-induced phase separation (VIPS) [174], followed by Elford’s development in 1937 [175]. Today, VIPS has become an important technology to produce polymer membranes. Compared to NIPS, the features of VIPS are that the non-solvent phase is a gas and the nonvolatile non-solvent is originally contained in the volatile solution, resulting in the non-solvent being enriched in the casting solution during the process of a controlled solvent evaporation. This implies that the phase separation is a process of a non-solvent intake rather than a solvent outflow. The polymer finally precipitates in the casting solution to form a membrane. Polymeric membranes fabricated by VIPS present the main advantage of possible morphology control with a relatively easy process. Therefore, the membranes prepared by VIPS are widely used for different applications. Porous membranes are used in water filtration [176,177,178,179,180,181,182], while dense membranes are usually applied in gas separation [183,184,185]. PVDF membranes are also used for proteins adsorption [186], while PS membranes could be efficiently used in membrane distillation [34]. However, commercial membranes produced by VIPS still remain limited in practice [187].

##### VIPS Processing

The process of flat-sheet membrane is as follows. Firstly, a homogeneous polymeric solution is made with appropriate solvent evaporation, then the solution is cast on a substrate at a desired initial thickness and placed in a VIPS chamber for the phase separation to occur. Subsequently, the polymer solution is immersed in a non-solvent bath and then the membrane is produced after a drying step. Some essential points must be controlled: (i) The conditions govern the degree of phase separation over the whole thickness, especially the exposure time to the non-solvent vapors and relative humidity. If a complete phase separation through the whole thickness is necessary, the immersion step is only a washing step. In case of VIPS/LIPS (liquid-induced phase separation), an uncomplete phase separation occurs, the non-solvent bath is set to not only remove all the solvent traces on the membrane, but also to induce phase separation to get a complete phase separation over the whole thickness. (ii) Ensure that the non-solvent inflow is higher than the solvent outflow during the phase separation of the casting solution. Note that hollow-fiber membranes also can be easily prepared via VIPS/LIPS [187].

##### Effect of Formulation Conditions on PS Membranes Morphology

(I) Effect of solvent and non-solvent composition

In VIPS, most cases use water vapors as a non-solvent due to its gas state and easy processing. Meanwhile, the choice of solvent is critical for membrane morphology and therefore physical properties. The solvent selection must be based on three key parameters: good solvency, appropriate viscosity and low volatility. Taking PS as an example, it can be seen that the N-methyl-2-pyrrolidone (NMP)/water system is the most commonly used, but DMF/water and 2-pyrrolidinone (2P)/water are also applied at smaller scale (see Table 6).

(II) Effect of polymer concentration

The polymer concentration effect on the morphology of PS membranes prepared by VIPS is among the main studies reported [183,187,189,197,198,199,200]. The results show that the polymer concentration can affect the viscosity of the system and therefore the resistance to non-solvent diffusion. Furthermore, the polymer volume fraction increases with the polymer concentration at the interface, resulting in the formation of a lower gravimetric porosity.

**Figure 5:**
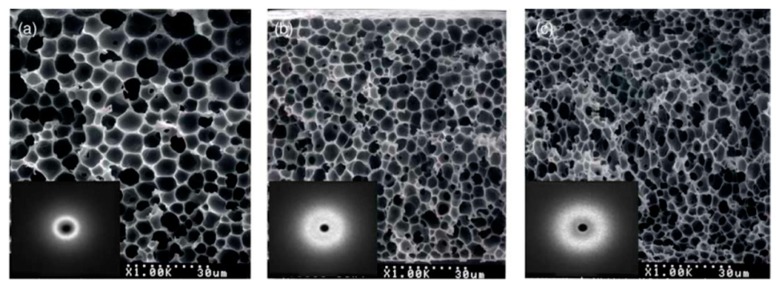
Effect of PS concentration on the membrane surface structure for different polymeric solution: (**a**) 15 wt.%, (**b**) 20 wt.% and (**c**) 25 wt.%, adapted from reference [190].

Su et al. [192] investigated two cases with different PS concentrations (10 wt.% and 20 wt.%) in the casting solution. It was found that the membrane morphology changed from a finger-like structure to a cellular-like structure with increasing polymer concentration because the demixing path was strongly dependent on the polymer concentration: spinodal decomposition occurred throughout the whole film thickness at 10 wt.% PS, while spinodal demixing was confined in a small region near the interface at higher PS concentration (20 wt.%). Lee et al. [190] also confirmed that the higher polymer concentration resulted in smaller pores structure (see Figure 5). Tsai et al. [76] showed that the polymer solution viscosity increased with an increasing polymer concentration. As a result, the polymer chains mobility was reduced as well as the non-solvent diffusion. Consequently, a higher resistance to the polymer-lean phase occurred, resulting in smaller pores.

(III) Effect of additives

Usually, the additives have hydrophilic groups such as PVP [193], and amphiphilic additives [196]. Their use increases the solution viscosity leading to lower solution mobility hindering the phase-separation kinetics but greatly enhancing the thermodynamics for the phase separation. Consequently, higher additives concentration led to increased membrane surface pore size, gravimetric porosity and pore interconnection, as well as the membrane pure water flux and antifouling property [194]. This means that the final membrane morphology could be tailored by the concentration of these additives leading to optimized membrane properties such as improved hydrophilicity, enhanced mechanical properties and increased gravimetric porosity. However, considering a trade-off between the thermodynamic instability and the rheological behavior of the systems, excessive additives content may lead to shrinkage, tearing or similar defects in the membrane formation [201].

##### Effect of Process Parameters on PS Membranes Morphology

(I) Effect of exposure time

**Figure 6:**
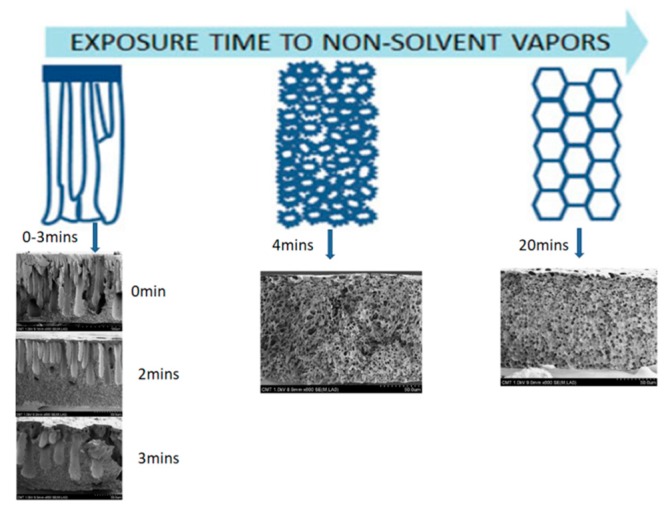
Membrane cross-section structure as a function of time that the cast film was exposed to humid air (20 wt.%, 25 °C and 70% relative humidity), adapted from references [187,192]. [A color figure can be viewed in the online issue, which is available at wileyonlinelibrary.com.].

Su et al. [192] used a PS solution (10 wt.% or 20 wt.%) into a chamber maintained at 25 °C and 70% relative humidity, to investigate the effect of the exposure time on membranes formation. The results indicated that increasing the exposure time led to the morphology going from finger-like macrovoids to sponge-like pores with a bicontinuous structure being an intermediate state (see Figure 6). Generally speaking, longer exposure time will lead to smaller droplet size of the polymer-lean phases and the further extent of the coarsening [189]. For too short exposure times to water vapors, an asymmetrical structure with a dense layer and finger-like macrovoids similar to a membrane directly immersed in water was obtained. For low exposure times, the structure is bi-continuous due to nucleation prevailing over growth. As the exposure time is increased, the pores sizes increase with growth of the polymer-lean phase, while coalescence of the polymer-rich phase is developed [187].

(II) Effect of relative humidity (RH)

As water is the most commonly used non-solvent in the formation of membranes by VIPS, the term non-solvent partial pressure will be associated to RH.

Park et al. [189] investigated the effect of RH on the morphologies of PS membranes by VIPS. When the PS 30 wt.% solution and the RH was in the range of 70%–100%, the average pore sizes varied from 11.8 μm to 5.5 μm and all cases produced a cellular-like structure with pore sizes decreasing with an increasing RH. A similar result was reported in the preparation of PS/NMP and PS/2P cast films [76]. These results highlighted the effect of different water chemical potential between the air and the system on the final membrane morphology.

From these studies, it can be concluded that with increasing RH from a certain value that is depending on the system, the growth of polymer-lean phase can be restrained but is concomitant to the higher driving force for non-solvent transfer, giving less time to the coarsening process of the polymer-lean phase to occur and therefore yielding smaller pores.

(III) Effect of air gap

**Figure 7:**
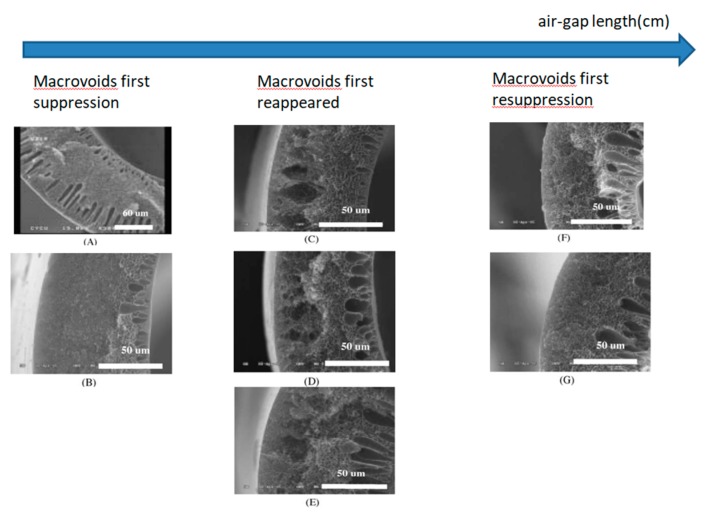
Membrane cross-section morphology of PS hollow fibers prepared with an ambient relative humidity of 30% for different air gap length (cm): (**A**) 0 cm, (**B**) 10 cm, (**C**) 20 cm, (**D**) 30 cm, (**E**) 40 cm, (**F**) 50 cm and (**G**) 60 cm, adapted from [191].

For PS hollow fiber membranes, the effect of air water vapor plays a key role on the morphology. Firstly, the influence of the air-gap length on the PS hollow fiber membranes morphology was investigated. Tsai et al. [191] kept all the parameters constant except for the air-gap length (0 cm, 10 cm, 20 cm, 30 cm, 40 cm, 50 cm and 60 cm). They observed than with increasing the air-gap length, macrovoids suppression occurred first, then macrovoids formation was observed before disappearing again. As shown in Figure 7, complete suppression of the macrovoids near the outer surface first occurred when the air gap was 10 cm and macrovoids can no longer be seen when the air gap was above 50 cm. So the morphology near the hollow fibers outer surface strongly depends on the air gap length. Furthermore, they proposed an explanation for the initial macrovoids suppression: the water vapor in the air gap was drawn to the dope. However, with longer air gap, the macrovoids reappeared due to the gelation of the polymer-rich phase disappearing if enough time was given for polymer relaxation. Finally, macrovoids resuppression occurred when the air gap was long enough to from a phase separation as the dope finally reaches the coagulation bath.

Next, Tsai et al. [191] investigated the effect of the air gap relative humidity on the morphology of hollow fiber membranes. The results of Table 7 show that when the relative humidity is high, the air-gap length required for the first suppression and the resuppression of macrovoids decreased because the water vapor in the air gap was drawn to the dope.

(IV) Effect of temperature

The VIPS chamber temperature and the dissolution temperature of the polymer may influence the rate of mass transfer and the demixing kinetics, but the effect of temperature on the membrane morphology does not seem to be as important as other parameters [201]. However, low temperature leads to lower mass transfer rates subsequently forming a polymer volume fraction gradient. This transformation results in different demixing paths, so its effect on the formation of polymeric membranes remains important [187].

### 4.2. Electrospinning

In 1914, Zeleny [203] conducted a detailed analysis of the electrospinning technique. From 1934 to 1944, Anton filed a series of patents for electrospun polymer filaments (US Patent Number: 2,116,942 [204], 2,160,962 [205] and 2,187,306 [206]). In recent years, the popularity of nanomaterials and nanotechnologies promoted the use of electrospinning which regained more and more attention. Since electrospinning has the ability of fabricating ultrafine fibers or various polymers fibrous structures with diameters ranging from micron to nanoscale, electrospinning has been widely used to prepare nano-fibrous membranes. These highly porous membranes have high gravimetric porosity, excellent pore interconnectivity, micron scaled interstitial space, low density, controllable thickness and a large surface area to volume ratio with exceptional mechanical strength [42,48,207,208]. These outstanding properties lead to various applications in separation membranes, affinity membranes, water and air filters, etc. [16,34,38,41].

Among the synthetic polymers used for electrospinning, PS and PVDF exhibit several attractive attributes in membrane fabrication, such as excellent chemical resistance (broad pH range, good chlorine tolerance), excellent mechanical strength, good solubility, good thermal stability, and good processability. Furthermore, polymer membranes have the advantages of low cost, efficiency, low tortuosity, high surface gravimetric porosity, and hydrophobicity [16,34,48]. So, for an electrospinning process, PS and PVDF can be considered to be model polymers [209].

#### 4.2.1. Electrospinning System and Process

Since electrospinning can be performed with various polymers both in solution and in the melt [210], a classification of the electrospinning techniques into solution electrospinning and melt electrospinning is made. Compared to solution electrospinning, some advantages of melt electrospinning are: (i) productivity as it can overcome technical limitations such as solvent selection as well as solvent accumulation and toxicity [211]; (ii) cleaner processing, environmental safety and health benefit (no requirement of polymer dissolution in organic solvents and their removal/recycling; (iii) less expensive, such as sub-micron scale fibers of polymers lacking appropriate solvents at room temperature can be fabricated with a higher throughput without mass loss by solvent evaporation [207]. Therefore, there is a great interest in producing fibers using melt electrospinning, such as PEG/PVDF core/shell nanofibers [212]. However, very little progress has been made in the past decades. At present, melt electrospinning is still in its infancy because of high viscosity, a very high processing temperature and its inability to produce fibers in the nanometer ranges. The higher temperatures may restrict their applications in the field of tissue engineering and drug delivery. Full understanding of this process and its potential to replace solution electrospinning has not been realized yet [207]. This is why a focus on solution electrospinning is presented next.

**Figure 8:**
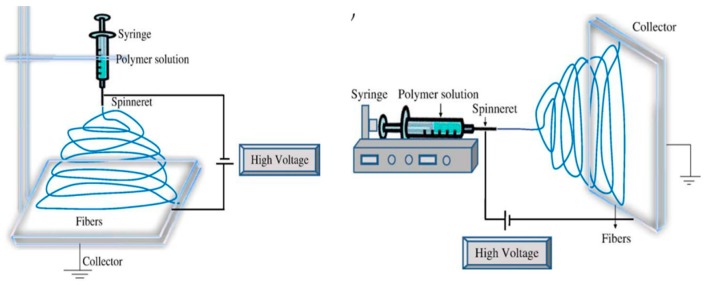
Typical set-up for electrospinning in the vertical (**left**) or horizontal (**right**) configuration, adapted from reference [207]. [A color figure can be viewed in the online issue, which is available at wileyonlinelibrary.com.].

Currently, a conventional solution electrospinning system consists of a high voltage power supply, one or more grounded collectors and a spinneret [207,208,213,214,215,216]. As shown in Figure 8, an electrospinning apparatus can be run vertically and horizontally at room temperature [207].

A typical electrospinning process runs as follows. Firstly, the polymer fluid, such as a melt or blend solution, is introduced into the capillary tube. Secondly, a strong electric field is applied between a spinneret and a grounded collector. When the applied voltage overcomes the surface tension of the polymer fluid, the strong electric field causes a droplet shape to deform into a conical structure (Taylor cone). Then, the charged polymer solution is ejected from the tip of the Taylor cone to the collector of opposite polarity. Furthermore, most of the solvent is evaporated due to an unstable whipping motion and continuous elongation via electrostatic repulsion occurring between the nozzle and the collector. Finally, solidification of the fluid jet forms an electrospun membrane [207,208,213].

#### 4.2.2. Effects of Various Parameters on Electrospinning

In this section, the control of PS membrane using the electrospinning method will be discussed at first, then some features of the PVDF electrospinning membranes are presented.

**Figure 9:**
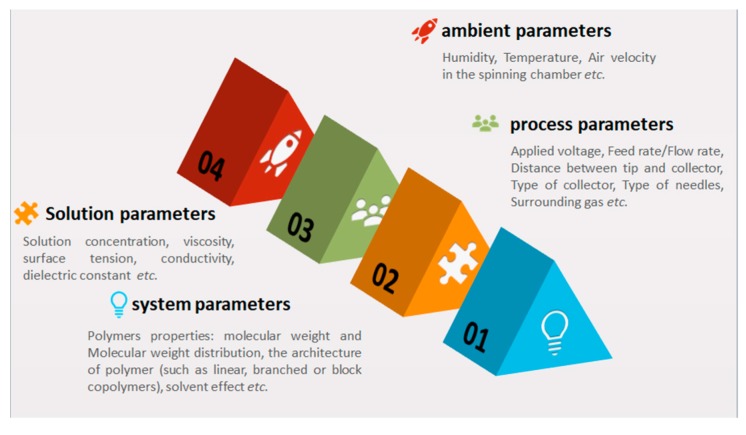
Classification of the main parameters influencing the electrospinning process, adapted from reference [48].

As reported in the literature [207,208,209], four key parameters are affecting the electrospinning process which can be classified into system, solution, process and ambient parameters (see Figure 9).

##### Effect of Solvent

The solvent has basically two important functions. Firstly, it helps to dissolve the polymer molecules and forms a solution to generate the electrified jet and secondly, it carries the dissolved polymer molecules to the collector [207,217]. In the dissolution step of electrospinning, the solvent evaporation rate and the polymer drying time depend on the solvent properties. It is critical for a successful process to select an appropriate solvent system. For PS membranes, some typical solvents have been used due to good volatility, vapor pressure and boiling point [207]. Typical examples are DMAC, DMF and acetone [207,208,209,213,214,215,216,218]. Moreover, as the solvent itself can interact with the electric field during the fiber formation, appropriate solvent polarity is important for fiber formation. Usually, a dipolar aprotic solvent has a high dielectric constant and dipole moment. Compared to the dielectric constant (25 °C) of dichloromethane (DCM) of 8.93, the value of DMF is 36.71. This means that DMF has higher solvent polarity [219]. Previous works confirmed that the addition of a dipolar aprotic solvent increased the solution conductivity which was a prerequisite for the formation of bead free uniform fibers [207]. Furthermore, some researchers mixed high polarity solvent with low polarity solvent to optimize the solvent polarity, solution viscosity and charge density of the polymer jet. The combined effects greatly contributed to producing good fibers [219].

##### Effect of Solution Viscosity, Surface Tension and Conductivity

The solution viscosity can significantly influence the generation of beads and their disappearance by increasing its value. Furthermore, higher viscosity forms larger fiber diameter. For surface tension, there is no obvious relation with the fiber morphology, but higher surface tension may results in jet instability [207]. Meanwhile, Yuan et al. [209] found that the polymer concentration contributes to the solution viscosity. On the other hand, surface tension seems to be more dependent on the solvent compositions than on the polymer concentration. The solution conductivity is mainly determined by the polymer type, solvent used, and the availability of ionizable salts. Zong et al. [220] reported the effect of higher solution conductivity by adding ionic salt on the morphology and diameter of electrospun fibers. They added ionic salts like KH_2_PO_4_, NaH_2_PO_4_, and NaCl to produce beadless fibers with relatively smaller diameters ranging from 200 to 1000 nm. Using salts, the fiber uniformity increased and beads generation decreased. It has been found that increasing the solution electrical conductivity, a significant decrease in the electrospun nanofibers diameter was observed, while lower solution conductivity resulted in insufficient elongation of the jet by electrical force to produce a uniform fiber leading also to bead formation [207].

##### Effect of Polymer Concentration

**Figure 10:**
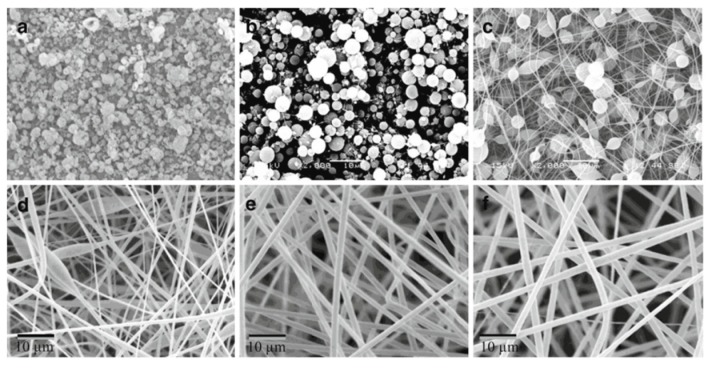
SEM micrographs of electrospun samples prepared using different PS concentrations in dimethylformamide (DMF) as solvent: (**a**) 5 wt.%, (**b**) 10 wt.%, (**c**) 15 wt.%, (**d**) 20 wt.%, (**e**) 25 wt.% and (**f**) 30 wt.%, adapted from reference [213].

As mentioned above, low polymer concentration will lead to low viscosity. Al-Qadhi et al. [213] showed that when high surface tension forces overcome the viscous forces (low viscosity), a bead structure on the PS surface was formed. The effect of solution concentration on the resulting morphology of the PS surfaces is clearly illustrated in Figure 10. Beads can be seen at 5 wt.% of PS, but a combination of microspheres and nanofibers structure was formed with increasing solution concentration to 10 wt.%, 15 wt.% and 20 wt.% of PS. Uniform and ultrafine fibers were prepared from solutions with 25 wt.% and 30 wt.% of PS. Meanwhile, the fiber diameter increased with increasing PS concentration as clearly illustrated in Table 8. Several studies found that the average fiber diameter increased with polymer concentration following a power-law with an exponent in the 2–3 range [209,213,214,221,222].

##### Types of Collectors and Needles

Several different types of needle shapes and collectors are available to control the morphology and alignment of complex nano-structures [48,207]. Electrospun highly aligned fibers with hierarchical features can be spun by using plate-type, drum-type, disc-type and counter electrode array-type collectors [48,223]. Among them, the plate-type collector is the most simple, versatile and widely used. The drum-type has been developed to fabricate large area fibers with high alignment. Also, the electrospun membranes can be fabricated using the disc-type collector and the counter electrode array-type collector, but their large-scale production are more difficult than others [48,223,224,225,226,227,228]. There are four types of nozzles for electrospinning: single nozzle, multi-nozzles, needleless and co-axial nozzles. The single nozzle is available for lab-scale production, while multi-nozzle is widely used for larger scale production due to its modularity. Needleless types can easily fabricate an electrospun membrane with a broad diameter distribution at high rate, while co-axial nozzles can be used to fabricate hollow nanofibers [48,223,228,229,230].

##### Effect of Applied Voltage

When the voltage is too low to overcome the solution surface tension, a fiber cannot be generated by the required elongation force. Conversely, excessive voltage leads to instability of the Taylor cone [213]. As mentioned previously, the required applied voltage to overcome the surface tension of the polymer solution can be calculated as:

(7) $$V_{c}^{2}=4\left(\frac{H^{2}}{L^{2}}\right)\left(\ln\frac{2L}{R}-1.5\right)\left(0.117\pi R\gamma \right)$$

where *H* (cm) is the distance between the tip and collector, *L* (cm) is the nozzle length, *R* (cm) is the nozzle radius and *γ* (dyne/cm) is the polymer solution surface tension [48,215,231,232,233]. In a typical electrospinning process, a high voltage in the range 5–30 kV is applied to the polymer solution [209]. When the applied voltage was controlled to reach a stable Taylor cone, the results of Yuan et al. [209] showed a slight tendency to produce smaller fiber diameters with increased voltage. In addition, Al-Qadhi et al. [213] noted that the optimum electrospinning voltage increases with the increasing of the PS concentration.

##### Effect of the Tip-Collector Distance

**Figure 11:**
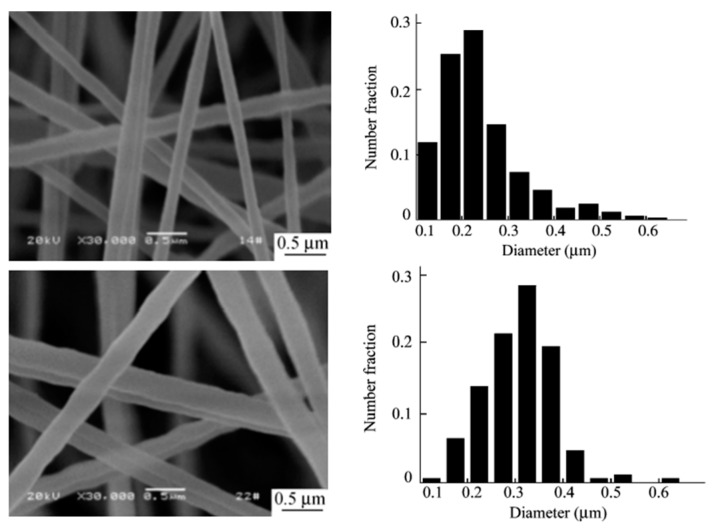
Effect of spinning distance (Top = 19 cm and Bottom = 15 cm) on the average fiber diameter and its distribution for PS nanofibers, adapted from reference [216].

According to Yao et al. [216], the average diameter of PS nanofibers decreased from 300 nm to 150 nm with increasing the distance between the tip and collector from 15 cm to 19 cm (see Figure 11) due to the longer tip-collector distance resulting in more solvent evaporating and the splitting degree of the jet flow increasing with distance. As a result, the diameter of the electrospun fibers decreased. Furthermore, Bhardwaj et al. [207] showed that if it was too short or too long distances were used, the morphologies of the PS electrospun fibers were obviously turned into bead shapes.

##### Effect of Feed Rate/Flow Rate

Yuan et al. [209] prepared electrospun PS fibers at 0.40 mL h^−1^ and 0.66 mL h^−1^ flow rates from a 20 wt.% PS/DMAC solution at 10 kV and 10 cm capillary-screen distance. The results indicated uniform ultrafine fibers when the flow rate was 0.40 mL h^−1^, but the fibers showed a bead-fiber morphology when the flow rate increased to 0.66 mL h^−1^. So high flow rates seem to promote the formation of a bead-fiber structure. However, low flow rates give more time for solvent evaporation, leading to thinner fibers [213].

##### Effect of Humidity

Huang et al. [218] found that high humidity led to larger fiber diameter. When the humidity was 0%, 10%, 20%, 30%, 40% and 50%, the average PS fiber diameter was 1.15 μm, 1.61 μm, 2.29 μm, 3.19 μm, 3.26 μm and 3.58 μm, respectively. The PS can dissolve very rapidly in the presence of water vapor, and hence, the PS fibers maintain their uniformity at lower humidity. However, very large and very small fibers can be formed at higher humidity. As a result, the fiber size distribution at high humidity is wider than the distribution at low humidity. Since the formation of nanopores on the fibers results in weaker PS fibers, humidity was also shown to have a direct effect on the mechanical properties of PS fiber mats: the electrospun fibers at low humidity had higher strength than those produced at high humidity [218].

It is important to note that the final polymer membrane morphology and performance are optimized by the combined effects of all these parameters. Similar to PS membranes, PVDF has good mechanical properties and excellent resistance to heat, abrasion and chemicals; this is why it is widely used for electrospinning [48]. Table 9 lists some examples of PVDF membranes produced via electrospinning to highlight the difference between the nanofiber layer and the total membrane layer. It can be seen that the PVDF membranes via electrospinning usually have a controlled pore structure and high gravimetric porosity. In addition, this method mostly leads to the β-phase PVDF over the other crystalline polymorphs due to its highest dielectric constant and polar property. Recently, some researches focused on using various kinds of additives in PVDF such as partially negative additives like fluorine-based fillers to interact with the partially positive hydrogen atoms in the PVDF chain; or using partially positive additives to interact with the partially negative CF_2_ groups in the PVDF chains [48,234,235,236,237,238,239].

### 4.3. Track Etching

#### 4.3.1. Swift Heavy-Ion Irradiation

**Figure 12:**
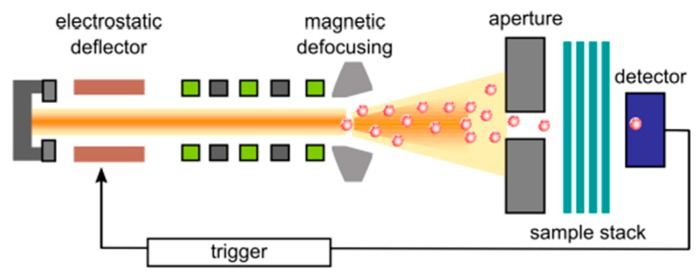
Schematic representation of a single-ion irradiation setup, adapted from reference [244]. [A color figure can be viewed in the online issue, which is available at wileyonlinelibrary.com.].

Swift heavy-ion beams are commonly provided by linear accelerator facilities and cyclotrons. These heavy ions (up to uranium) beams of high energy have a penetration range in polymers of about 120 µm, so foil stacks can be irradiated. Each ionic projectile induces electronic excitation and ionization in a cylindrical zone along its trajectory. For polymers such as polyimide (PI), polyethylene terephthalate (PET), polycarbonate (PC) and PVDF, the chemical bonds are commonly damaged and hence small volatile fragments (H_2_, CO, CO_2_, hydrocarbons) are easily outgassed [243]. As a result, some nanometers pores are produced. Meanwhile, the destroyed regions are defined as the ion track. A typical schematic representation of the single-ion irradiation setup is presented in Figure 12. The system includes: electrostatic deflector, magnetic defocusing instrument, detector, trigger and sample stack. Firstly, the ion beam is strongly defocused and adjusted, then single projectiles pass through a small circular aperture (diameter ≈ 200 μm) with a frequency of about 1 Hz. Next, the ion beam irradiates a stack of foils. If a solid-state particle detector is placed behind the sample, it will register a single ion impact and the entire ion beam will be deflected by an electrostatic chopper system. Furthermore, the gravimetric porosity regime is available by using ion-track technology: single channel, non-overlapping channels, and overlapping channels by means of increasing ion fluence [244].

Generally, the production of porous membranes with uniform pore size requires continuous and homogeneous damage along the ion trajectory. The best results are achieved when the energy loss of heavy-ion projectiles (Au, Pb, Bi, U) in the given materials is above the etching threshold. Inhomogeneous and absence of etching can be obtained by decreasing the energy loss of the ions, resulting in pores with a broad size distribution [245].

Poly (vinylidene fluoride) (PVDF) is one of the most attractive polymers commercially available due to its biocompatibility, good resistance against high temperature and excellent chemical stability. In particular, PVDF in the β-phase has special properties such as piezo/pyroelectric properties. Due to these interesting technological features, some researchers focused on the production of track-etched PVDF membranes using swift heavy-ion irradiation and subsequent chemical etching. The main results are presented in Table 10. The production of track-etched membranes with pore diameter in the nanometer or micrometer scale has been used in a variety of growing technological fields such as fuel cells [246].

#### 4.3.2. Chemical Etching

Exposure of ion-irradiated PVDF membranes to a suitable etching solution can selectively dissolved the ion tracks and be subsequently enlarged into channels [244,255]. The etching conditions, such as etchant composition and etching temperature, play critical roles on the channels morphology. In addition, ion-irradiated PVDF membranes heated in air prior to chemical etching leads to a more uniform pore size of the channels [246,252].

##### Effect of the Etchant Composition

Komaki exposed PVDF membranes to fission fragments in oxygen and etched the film in sodium hydroxide and potassium hydroxide solutions [249]. Zhao et al. [250] obtained an optimum etchant of KMnO_4_ in KOH. Grasselli and Betz reported that different etching conditions involving permanganate oxidation in different alkaline environments could be tuned to prepare track-etched PVDF membranes with a desired pore diameter [248]. However, the severe etching conditions with a highly concentrated aqueous KOH solution in addition to KMnO_4_ in most cases damaged the whole membrane, imposing particularly irreversible chemical damage on a non-irradiated part. As a result, some distinctive properties of the PVDF membranes were destroyed [252]. Consequently, moderate etching conditions, such as a pure alkaline solution at high temperature without any oxidant additives, gained more popularity in recent years [252,254].

##### Effect of Etching Temperature

**Figure 13:**
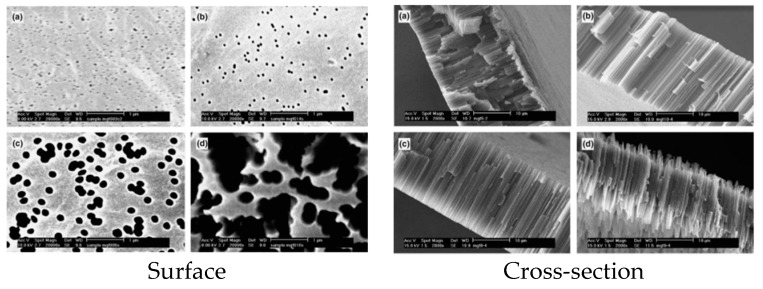
Scanning electron micrograph of irradiated PVDF films etched with KMnO_4_ 0.25 mol L^−1^ solution in KOH 9 mol L^−1^ at: (a) 55 °C, (b) 65 °C, (c) 75 °C and (d) 85 °C. The fluence is 4.7 × 10^9^ cm^−2^ (55 °C) and 9.5 × 10^8^ cm^−2^ for the other conditions. Etching time is (**Left**) 1 h and (**Right**) 3 h, adapted from reference [248].

Grasselli et al. [248] studied the etching temperature effect on the diameters and shapes of the surface and cross-section pores for Sn irradiated PVDF membranes. Figure 13 shows that the average pore diameter increases from 82 to 202 and 398 nm when the etching temperature increased from 55 to 65 and 75 °C, respectively. On the other hand, the cross-section view of the track-etched PVDF foils showed that it was possible to control the pore size and structure by changing the temperature of the etching process. For a temperature up to 75 °C, the pore structures were straight, cylindrical and open, while further temperature increase to 85 °C produced clearly modified pore shapes, mainly a conical pore aperture over the first few micrometers of each surface.

##### Effect of Etching Time

Grasselli et al. [248] found that the pore diameter increased rapidly in the first 15 min, then 1 h later the diameters stabilized, hence the rate of etching was 30, 80, 100 and 100 nm/h at 55, 65, 75 and 85 °C, respectively. As a conclusion, the kinetic behavior was simultaneously affected by the etching time and temperature.

### 4.4. Sintering

Sintering involves the process of a given set of particles being compacted first to transform them into a physically robust and dense polycrystalline monolith at elevated temperatures [46,256]. This method is widely used in the commercial production of inorganic membranes and some polymer membranes (symmetric polytetrafluoroethylene and polypropylene). For the production of porous PVDF membranes via sintering, Dickey and Mcdaniel were granted a patent (US3896196A) in 1975 [257]. They dispersed a PVDF powder in methyl isobutyl ketone (MIBK) and then the dispersion was broken into droplets. Sintering of the droplets at specific temperatures occurred and finally, a porous PVDF structure was prepared by sintering. In 1984, Georlette and Leva obtained cellular or dense structures by the extrusion of a molding composition comprising of a vinylidene fluoride polymer and a blowing agent: hydroxycarboxylic acid or an alkali metal salt derived from one of the acids [258]. However, due to the slow solid state diffusional processes, in most cases the sintering transformation is driven by temperatures between 1/2 and 3/4 of the melting point. These high temperatures result in limited material degradation, material synthesis, phase stability and high processing cost, especially for incompatible and co-sintering thermoplastic polymers.

**Figure 14:**
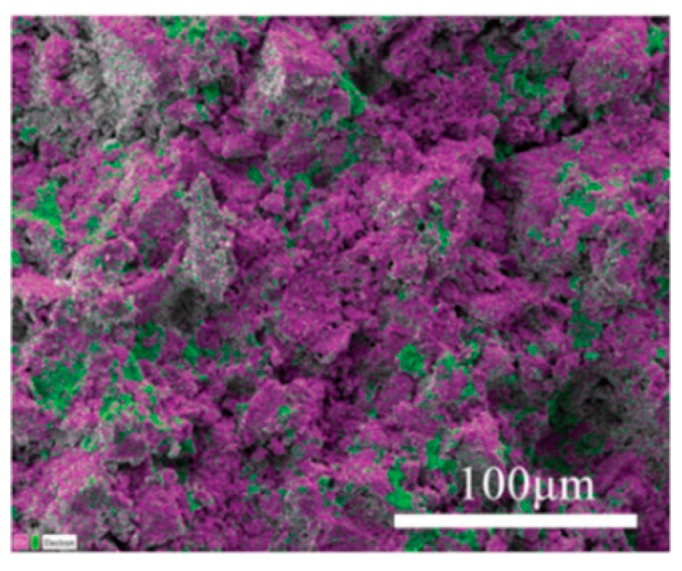
Energy dispersive spectroscopy (EDS) map superimposed on back scattered image of 80LAGP–20(PVDF-HFP) cold co-sintered at 120 °C and 400 MPa for 1 h before soaking in 1 M LiPF_6_ ethylene carbonate-dimethyl carbonate (EC-DMC) (50:50 vol.%). Elemental Ge is shown in purple and elemental F is shown in green, adapted from reference [256].

Today, most techniques are explored to push the temperature window into the desired low temperature range to perform cold sintering process (CSP) since the active temperature window spans the range from room temperature to 200 °C [256,259]. Guo et al. [256] used (1−x)LAGP–x(PVDF−HFP) (x = 0, 5 or 10 vol.%) powder as the raw material for the composite cold sintering, 30–39 vol.% of deionized water was added to LAGP and homogenized in a mortar and pestle, then the samples were left under 400 MPa of uniaxial pressure and 120 °C for 1 h. The results showed that the thermoplastic polymers and ceramic materials could be jointly formed into dense composites under these conditions (see Figure 14). Moreover, the dense composites have improved microwave, dielectric and ionic electronic transport. The CSP method bridges the gap between the sintering of traditionally incompatible material systems, such as ceramics and thermoplastic polymers, by opening up a novel and effective route to develop the field of multifunctional material manufacturing [256,259,260].

### 4.5. Comparison between the Preparation Methods

Table 11 presents an overview of the works done using different processes to produce porous polymeric membranes.

## 5. Conclusions

In this review, a basic description about the preparation and characterization of porous polymeric membranes was given. In particular, the two main synthetic polymers used for membranes production have been discussed into details: polysulfone (PS) and polyvinylidene fluoride (PVDF). Five different methods for membrane fabrication were introduced: non-solvent induced phase separation (NIPS), vapor-induced phase separation (VIPS), electrospinning, track etching and sintering. The basic principles of each process and the formation mechanisms for the membranes were discussed in terms of their advantages and limitations. This included key factors such as the system, solution, processing and ambient parameters. Based on all the possibilities available, it is expected that porous polymeric membranes with well-controlled morphology, good stability, and excellent permeation properties can be obtained to develop more applications in membranes technologies.

## Figures and Tables

**Table 1:** Typical Properties of PVDF [46].

Properties	Range
Crystallinity (%)	35 to 70
Melting point (°C)	140 to 170
Glass transition temperature (°C)	−41 to −38

**Table 2:** Examples of different solvent/non-solvent systems for PS membrane fabrication.

Solvent/Non-Solvent System	Casting Solution (wt.%)		Parameters of the Solvent						Refs.
	PS	Solvent	δ_d_ *	δ_p_ *	δ_h_ *	R_o_ *	R_a_ *	RED	
N-methyl-2-pyrrolidone (NMP)/water	10.62–18	75–83	18	12.3	7.2	-	5.4	0.7	[43,80]
N,N-dimethylacetamide (DMAc)/water	5–15	85–90	16.8	11.5	10.2	-	6.9	0.9	[81]
NMP, tetrahydrofuran (THF)/water	21	NMP/THF = 70/30	19	10.2	3.7	-	5.2	0.6	[82]
N,N-dimethylformamide (DMF)/water	16	83	-	-	-	-	-	-	[38]

* Unit of δ_d_, δ_p_, δ_h_, R_o_, R_a_ is MPa^1/2^.

**Table 3:** Examples of supercritical carbon dioxide (sc-CO_2_) use as a non-solvent in the preparation of PVDF membranes.

Polymer	Solvent	Additive	Experimental Condition	Pore Structure	Ref.
PVDF-HFP	acetone		Polymer concentration: 10–25 wt.%; CO_2_ pressure: 8.5, 13.5 and 18.5 MPa; Temperature: 35, 45 °C	“sponge-like” and asymmetric structure.	[92]
PVDF	DMAc	PMMA	Polymer concentration: 7.5–20 wt.%; CO_2_ pressure: 10 and 20 MPa; Temperature: 45, 65 °C PMMA concentration: 1.5, 4.5 and 7.5 wt.%	an asymmetric structure with cellular pores surrounded by interlinked PVDF particulate crystallites	[93]
PVDF–HFP	acetone		Polymer concentration: 1–20 wt.%; CO_2_ pressure: 80–200 bar; Temperature: 35–55 °C	cellular structure; bicontinuous structures; a leafy-like sub-morphology	[85]
PVDF–HFP	DMAc, DMF, NMP, TEP, acetone		Polymer concentration: 15 wt.%; CO_2_ pressure: 13.5 MPa; Temperature: 35 °C	“sponge-like” and asymmetric structure	[85]

PVDF-HFP = poly (vinylidene fluoride-co-hexafluoropropylene); DMAc= N,N-dimethylacetamide; DMF is dimethylformamide; NMP = N-methyl-2-pyrrolidone; TEP is triethylphosphate; PMMA is poly(methyl methacrylate).

**Table 4:** Typical additives used for PS membrane fabrication with their conditions.

PS	Solvent/Non-Solvent	Additive	Morphology		Configuration	Ref.
PS (Mw = 30 kDa)	1. NMP/water 2. DMAc/water	PVP (Mw = 24, 40 and 360 kDa)	Pore density	PS/NMP/PVP: 2.6 × 10^9^–9.8 × 10^9^	Flat-sheet	[43]
				PS/DMAc/PVP: 5.5 × 10^9^–10.5 × 10^9^		
			Pore size (nm)	PS/NMP/PVP: 3.0–3.62		
				PS/DMAc/PVP: 3.21–3.88		
PS (Udel 1700)	DMAc/water	PVP (K15, Janssen Chemical)	Flat-sheet: macrovoids. hollow fibers: large pores on the external surface with a spongy structure underneath.		Flat-sheet and hollow fibers	[128]
PS (Mn = 26 kDa)	NMP/water	1. PEG, (Mn = 8 kDa) 2. PVP (Mn = 8 and 40 kDa)	Pore size (nm)	PS: 59–61.8	Flat-sheet	[129]
				PS+PEG: 113.8		
				PS+PVP: 60.6–75		
PS (Ultrason S6010)	NMP/water	Tetronic- 1107	Pore size (nm)	7–13	Flat-sheet	[111]
PS (Mw = 77–83 kDa)	NMP/water	1. graphene oxide (GO) nanosheets 2. Janus graphene oxide (Janus GO) nanosheets	Pore size (nm):	PS: 11.21	Flat-sheet	[80]
				GO/PS: 15.82–19.67		
				JanusGO/PS: 18.50–21.01		
			Thickness (μm):	200		
PS	1. NMP/water 2. DMAc/water	polyethylene glycol (PEG), TiO_2_	Molecular weight cut-off (MWCO)	<600 Da	Flat-sheet	[81]
PS (Ultrason S 6010)	DMF/water	1. SiO_2_ nanoparticle 2. Polydopamine (PDA)	Root-mean-square (RMS) (nm)	PS: ~30	Flat-sheet	[38]
				PS/PDA: ~210		
				PS/SiO_2_: ~92		
				PS/SiO_2_/PDA: ~89		
PS (Mn = 22 kDa)	DMAc/water	1.Zn, Al-NO_3_LDH; 2. Trimethylsilylchlorosulfonate	Thickness (μm):	100	Flat-sheet	[39]
PS (M_W_ = 75 kDa)	NMP/water	1. PEG (M_W_ = 4 kDa); 2. Al_2_O_3_ nanoparticle	RMS (nm)	PS: ~69	Flat-sheet	[40]
				PS/Al_2_O_3_: ~96–119		
PS (Mw = 58 kDa)	DMAc/water	1. Reduced graphene (rGO)	Thickness (μm)	CMPS: 20–25	Flat-sheet	[33]
				CMPS/rGO: 22–30		

**Table 5:** Effects of various parameters on PVDF membrane morphology via NIPS.

System		Factor	Membrane Structure	Refs.
Solvent		weak solvent power	sponge-like	[77]
		stronger solvent power	Macrovoids	[78]
Non-solvent		weak non-solvent	Symmetric membrane consists of uniform spherical particles	[135,136]
		strong non-solvent	asymmetric structure consists of dense skin layer accompanied by finger-like or/and sponge-like structure	[136,137,138]
Coagulation bath temperature		high temperature	finger-like	[77,139]
		low temperature	sponge-like structure or/and particles (if crystallization occurs)	
Additives	Inorganic salts	low concentration	larger cavities and hence increase of gravimetric porosity and maximum pore size	[140,141]
		higher concentrations up to a certain value	less macrovoids formation	[142,143]
	Polymeric additives	PVP	more large finger-like macrovoids, higher gravimetric porosity and mean pore size	[136,137,138]
		PEG	higher pure water flux with a relatively lower rejection rate of membranes	[144,145]
	Non-solvent additives	water	larger pore radius and effective gravimetric porosity	[146]
		1,2-ethanediol	larger gravimetric porosity and pore size becomes more uneven	[147]

**Table 6:** Examples of PS membranes produced by VIPS.

PS (wt.%)	Additive	Solvent	Gaseous Non-Solvent	Process	Form	Ref.
15		DMF	Water	VIPS	Flat-sheet	[188]
17		NMP	Water	VIPS; VIPS/LIPS	Flat-sheet	[185]
15 and 30		NMP	Water	VIPS	Flat-sheet	[189]
15, 20 and 25		NMP	Water	VIPS	Flat-sheet	[190]
26		NMP	Water	VIPS	Hollow fiber	[191]
10 and 20		NMP	Water	VIPS; VIPS/LIPS	Flat-sheet	[192]
20		NMP	Water	VIPS/LIPS	Flat-sheet	[76]
12		2P	Water	VIPS/LIPS	Flat-sheet	[76]
15	PVP	NMP	Water		Flat-sheet	[193]
15	PVP/PANI	NMP	Water	VIPS/LIPS	Flat-sheet	[194]
20	Pluronic F108	NMP	Water		Flat-sheet	[195]
20	Pluronic F108, PluronicF127,3-DMMSAPS	NMP	Water		Flat-sheet	[196]

PVP = polyvinylpyrrolidone; PANI = polyaniline; DMMSAPS = (N,N-dimethylmyristylammonio) propane-sulfonate; Pluronic F = a triblock poly(ethylene oxide) − poly(propylene oxide) − poly(ethylene oxide) (PEO-PPO-PEO) copolymer; 2P = 2-pyrrolidinone.

**Table 7:** Relationship between macrovoids and air gap under different relative humidity [202].

Relative Humidity (%)	Macrovoids First Suppression (cm)	Macrovoids Reappeared (cm)	Macrovoids Resuppression (cm)
30	10	20	50
70	10	20	40
90	5	10	30

**Table 8:** Effect of PS concentration on the average fiber diameter [213].

	PS concentration (wt.%)	5	10	15	20	25	30
Diameter							
Bead size (μm)		2.5	2.9	3.6	2.5		
Fiber size (nm)			70	300	640	1200	1850

**Table 9:** Examples of PVDF membranes produced via electrospinning.

Ref	Samples	Crystalline Forms	Mean Pore Size (nm)	Nano- Fiber Size (nm)	Total Thickness (μm)	Total Porosity (%)	Nano- Fiber Layer Thickness (μm)	Nano- Fiber Layer Porosity (%)
[238]	Coaxial PPESK/PVDF	β-phase	-	-	45	75	-	-
	PPESK/PVDF at 160 ℃	β-phase	-	-	45	15	-	-
	PPESK/PVDF at 170 ℃	β-phase	-	-	45	4	-	-
[239]	Neat PVDF	β/α = 2.12	-	80–700	-	-	-	-
	As-cast PVDF	β/α = 3.24	-	80–700	-	-	-	-
	As-deposited PVDF	β/α = 3.79	-	80–700	-	-	-	-
	As-deposited PVDF with vertrel	β/α = 7.69	-	80–700	-	-	-	-
[240]	Neat PVDF	-	1.556	-	-	-	-	-
	PVDF/Chitin nanowhiskers (0.5 wt.%)	-	2.157	-	-	-	-	-
	PVDF/Chitin nanowhiskers (1.0 wt.%)	-	2.405	-	-	-	-	-
[241]	PVDF/Polyester (0.67 g/cm^3^)	-	1690	-	225 ± 15	69.7 ± 1.5	98 ± 10	89.4 ± 1.3
	PVDF/Polyester (0.64 g/cm^3^)	-	2010	-	331 ± 11	62.7 ± 1.2	122 ± 8	91.2 ± 1.2
	PVDF/Polypropylene (0.41 g/cm^3^)	-	1870	-	314 ± 18	67.9 ± 1.3	87 ± 11	88.8 ± 1.0
	PVDF/Polyester (0.50 g/cm^3^)	-	1650	-	190 ± 13	70.1 ± 1.5	103 ± 10	88.6 ± 1.2
[242]	Neat PVDF	-	-	91.28	470	98.77	-	-
	PVDF-PVP	-	-	108.09	580	97.59	-	-
	PVDF-AC	-	-	101.26	560	98.90	-	-
	PVDF- MnO_2_	-	-	102.14	560	97.62	-	-
	PVDF-PVP-AC	-	-	106.37	570	98.59	-	-
	PVDF-PVP- MnO_2_	-	-	105.01	570	97.88	-	-

**Table 10:** Examples of track-etched PVDF membranes using swift heavy-ion irradiation and subsequent chemical etching.

Swift Heavy-Ion Irradiation	Chemical Etching	Pre/Post-Treatment	Morphology	Ref.
Accelerator facility: van de Graaff (absorbed dose of 100 kGy) Cyclotron: GANIL (Caen, France) Energy (MeV/amu): 10.37 Fluence: 10^7^ and 5 × 10^8^ cm^−2^ Heavier projectiles: Kr under He atmosphere	Temperature (°C): 65; Time (h): 0.5–3; Composition: permanganate solution (0.25 M) in KOH (10 M)	Post-treatment: Radiografting	Structure: cylindrical pores; Pore size: 20–50 nm	[247]
Cyclotron: GANIL (Caen, France) Energy (MeV/amu): 2.85 Fluence: 9.5 × 10^8^ cm^−2^ and 9.5 × 10^9^ cm^−2^; Heavier projectiles: Sn in vacuum	Temperature (°C): 55–85; Time (h): 0.5–3; Composition: a saturated KMnO_4_ solution (0.25 mol L^−1^) prepared in different alkaline conditions (0.1, 0.9, 9 molL^−1^ KOH; 9 molL^−1^ KOH+ 0.004 mmol L^−1^ TBAm);	/	Structure: cylindrical open pores; conical-shaped pores; Pore size: <114 ± 18 nm	[248]
Thermal neutron flux: 1.7 × 10^13^ cm^−2^ s^−1^; Heavier projectiles: ^235^U with oxygen	Temperature(°C): <60; Time (h): 0–400; Composition: 5, 7.5, 10 and 12 mol L^−1^ KOH; 5, 7.5, 10 and 12 mol L^−1^ NaOH with ethanol and fluorochemical surfactant; 5, 7.5, 10 and 12 mol L^−1^ LiOH	/	Pore size: 30–220 nm Pore density (cm^−2^): 3 × 10^8^	[249]
Accelerator facility: GSI (Darmstadt, Germany); Energy (MeV/N): 13.7; Heavier projectiles: ^238^U	Temperature (°C): 70; Time (h): 0–50; Composition: KMnO_4_ (0.1, 0.15, 0.25 FW) in KOH (6, 7.5, 8, 10 N)	/	Pore size: <5 μm	[250]
Cyclotron: GANIL (Caen, France) Energy (MeV/amu): 10.37 Fluence: 10^7^–10^10^ cm^−2^; Heavier projectiles: Kr in a He atmosphere	Temperature (°C): 65; Time (h): 0.5; Composition: permanganate solution (0.25 M) in KOH (10 M)	Post-treatment: Radio-grafting	Pore size: 40 nm	[251]
Cyclotron: R7M Energy (MeV/amu): 41.6 Fluence: 10^7^–10^10^ cm^−2^; Heavier projectiles: ^40^Ar in a vacuum	Temperature (°C): 93; Time (h): 1–6; Composition: KMnO_4_ (2 gmL^−1^) in NaOH (6 N)	Pre-treatment: periodic heating of test samples	Pore size: 250 nm Pore density (cm^−2^): 1.8 × 10^9^	[246]
Accelerator facility: GSI (Darmstadt, Germany); Energy (MeV/N): 11.1 (^208^Pb), 6.7 (^58^Ni), 6.2 (^84^Kr) and 3.5 (^129^Xe); Fluence: 3 × 10^6^–3 × 10^11^ cm^−2^; Heavier projectiles: ^208^Pb, ^58^Ni, ^84^Kr and ^129^Xe	Temperature (°C): 80; Composition: KOH (9 mol dm^−3^)	Pre-treatment: periodic heating of test samples	Pore size: (nm) 305 ± 31 (^208^Pb), 118 ± 11 (^58^Ni), 109 ± 14 (^84^Kr), 93 ± 8 (^129^Xe)	[252]
Energy (MeV): 220; Fluence: 10^11^ cm^−2^; Heavier projectiles: Ar^9+^ in a vacuum	Temperature (°C): 90; Composition: KOH (10 N), KMnO_4_ (0.25 N) and KIO (0.5 N)	/	/	[253]
Energy (MeV): 450; Fluence: 3 × 10^7^ cm^−2^; Heavier projectiles: ^129^Xe in a vacuum	Temperature (°C): 80; Time: 0–50 h; Composition: KOH (9 mol dm^−3^)	/	Pore size: 25–265 nm	[254]

TBAm = Tetrabutylammonium bromide; KOH = potassium hydroxide; NaOH = sodium hydroxide; LiOH = lithium hydroxide; KMnO_4_ = potassium permanganate.

**Table 11:** Comparison of the various production methods presented.

Method	Process Steps	Pore Formation Mechanism	Description of Membrane Morphology	Advantages	Disadvantages
NIPS	Mixing, phase separation, solidification, final membrane.	Resulting from liquid-liquid phase demixing.	Usually with a wider pore size distribution and weaker mechanical properties than TIPS membranes.	NIPS can effectively control the pore size and other surface characteristics of the membranes with the help of additives.	The slow rate of mass transfer and the instability of the polymer/solvent dope at the interface of solvent and non-solvent, resulting in difficult precise control of the phase inversion process.
VIPS	Polymer solution with appropriate solvent evaporation, immersion and drying step, final membrane.	Resulting from the transfer at the interface, non-solvent (gas) inflow and solvent outflow.	A broad variety of morphologies, such as cellular-like, nodular-like and bi-continuous structure can be obtained and well controlled.	1. Slower mass transfer than wet-immersion enables modifying and tailoring the membrane morphologies. 2. Both flat-sheet and hollow-fiber polymer membranes can be easily prepared.	The development of commercial polymer membranes by the VIPS process still remains limited.
Electro- spinning	Polymer solution/melt, electrospun fibers, solvent evaporated and solidification, final membrane.	Resulting from the evaporation of the diluent.	Electrospinning enables the formation of interconnected pores with uniform pore size and porosities exceeding 90% in membrane.	1. Directly produce superhydrophobic polymer membranes. 2. Simple, inexpensive and high productivity. 3. Enables the production of highly porous structures of smooth non-woven nanofibers with high surface area to volume ratio and tunable gravimetric porosity.	Limited production capacity and low reproducibility.
Track etching	Swift heavy-ion irradiation, chemical etching, final membrane.	Irradiation produces latent tracks in the foils and pore formation via chemical etching.	Pore shape can be made cylindrical, conical, funnel-like or cigar-like and pore size can be easily varied.	The membrane pore size, shape and density can be precisely determined in a controllable manner.	1. Many large-scale applications are “insensitive” to track membranes, furthermore, it is cost extensive. 2. Track membranes are limited for some particular uses like proteins adsorption.
Sintering	The formation of polymer droplets, sintering of the droplets, final membrane.	Resulting from the sintering transformation driven by high temperatures.	Pore size can be controlled by the sintering process.	Widely used in the commercial production of inorganic membranes and some polymer membranes.	1. Sintering at specific high temperatures results in the limitation of material integration, material synthesis and phase stability. 2. High cost for processing.
